# Lepra Reaction with Lucio Phenomenon Mimicking Cutaneous Vasculitis

**DOI:** 10.1155/2014/641989

**Published:** 2014-12-17

**Authors:** Durga Prasanna Misra, Jyoti Ranjan Parida, Abhra Chandra Chowdhury, Krushna Chandra Pani, Niraj Kumari, Narendra Krishnani, Vikas Agarwal

**Affiliations:** ^1^Department of Clinical Immunology, Sanjay Gandhi Postgraduate Institute of Medical Sciences, Lucknow 226014, India; ^2^Department of Pathology, Sanjay Gandhi Postgraduate Institute of Medical Sciences, Lucknow 226014, India

## Abstract

Leprosy is a disease typically found in the tropics. Patients with leprosy can have varying presentation with constitutional symptoms, joint pains, skin nodules, and rarely a vasculitis-like picture with skin ulcers and neuropathy. We present a young lady who presented with the rare manifestation of skin infarcts mimicking cutaneous vasculitis, diagnosed on histopathology to have Lucio phenomenon on a background of lepromatous leprosy. With increasing migration and widespread use of biologic response modifiers, clinicians all over the world need to be aware of various presentations of leprosy as well as needing to keep an open mind while considering the differential diagnoses of vasculitis.

## 1. Introduction 

Leprosy refers to systemic infection caused by* Mycobacterium leprae*, or less commonly* Mycobacterium lepromatosis*. Only the former has been reported from India. Although endemic to the tropics, it is increasingly being found in developed countries outside of the tropical regions [1, 2], predominantly due to activation of latent infection in the context of immunosuppression with biologic response modifiers. This serves as a reminder of the global importance of this problem at a time when boundaries are shrinking [3] and widespread use of biologics is becoming the norm rather than the exception in the treatment of many immune-mediated diseases, including ankylosing spondylitis and rheumatoid arthritis.

Patients with leprosy can present with symptoms varying from constitutional to arthralgias and arthritis, mononeuritis multiplex, or frank lepra reactions [4, 5]. These can mimic a wide variety of common conditions including rheumatoid arthritis, lupus, and vasculitis [6]. We present a young lady who presented with large cutaneous infarcts that on the first impression were vasculitic but were subsequently proven to be due to Lucio phenomenon in the context of lepromatous leprosy.

## 2. Case Presentation

A 20-year-old lady presented with history of multiple nodular skin lesions, which were erythematous and were associated with stinging pain, 1-2 cm in size over both the upper and lower limbs and face for the past 1 year. This was associated with a low grade fever, on and off, responsive to antipyretic agents, for the same duration. She had history of pain in both knees at the onset of illness, for a period of 3 months, not associated with swelling, early morning stiffness, or pain in other joints, which was worse during the times she had fever. She had no dryness of eyes or mouth, tingling or numbness of extremities, shortness of breath, cough, chest pain, nasal or ear discharge, epistaxis, hearing loss, abdominal pain, weight loss, diarrhea, or dysuria. She had no foot drop or redness of eyes. She was investigated and found to have anemia (hemoglobin (Hb) 9.9 g%), normal total leucocyte count ((TLC) 6200/mm^3^), differential leucocyte count ((DLC) neutrophils 50%, lymphocytes 46%) and platelet count ((Plt), 261000/mm^3^), elevated erythrocyte sedimentation rate ((ESR), 36 mm/hour), and positive rheumatoid factor (RF) in serum by ELISA (26.11 IU, reference 0–15 IU). With this, she was thought to have rheumatoid arthritis and started on methotrexate 5 mg/week, hydroxychloroquine sulfate 200 mg daily, and methylprednisolone 4 mg daily. Subsequently, the skin lesion, fever, and joint pains subsided.

Three months later, while on the above-mentioned medications, the fever and skin lesions recurred and were of a similar nature and distribution as before. She now consulted a dermatologist who investigated and detected a persisting anemia (Hb 10.4 g%), mild leukocytosis (TLC 11230/mm^3^, DLC showing neutrophils 69%, lymphocytes 23%), normal platelet count (295000/mm^3^), and ESR elevation of 99 mm/hr. On the basis of her symptoms, she was diagnosed to have type II lepra reaction (erythema nodosum leprosum (ENL)) and started on prednisolone 60 mg/day and antileprotic therapy with rifampicin 600 mg/month, clofazimine 300 mg/month and 50 mg/day, dapsone 100 mg/day, and ofloxacin. There was a transient relief of symptoms, but these again recurred. As a consequence she visited multiple physicians over the next 4 months without avail, while continuing the same antileprotic drugs.

A week prior to presenting to us, she developed additional similar skin lesions over the trunk, along with blackish discolouration over the skin lesions on the face, legs, and dorsum of feet. 2 days prior to presentation, she developed pain and swelling of dorsa of both feet and ankles. Review of her past history and family history were insignificant for any diagnoses of leprosy.

**Figure 1:**
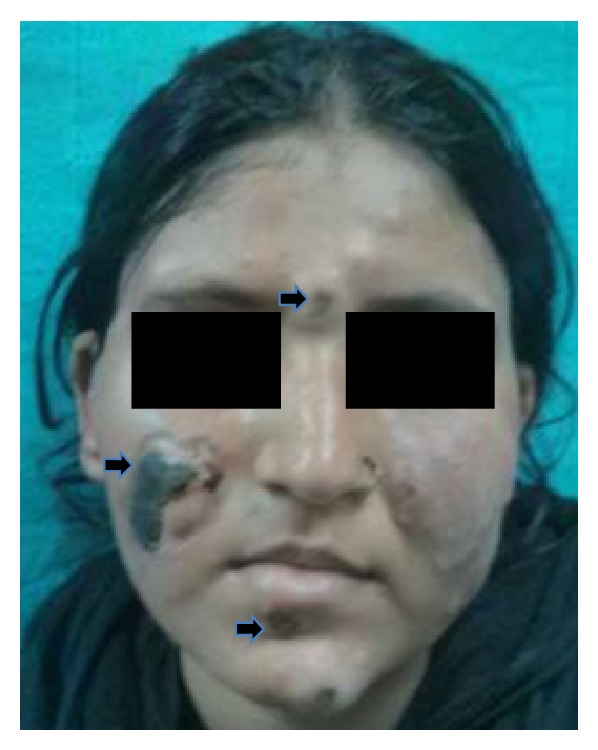
Image of face showing papulonodular lesions over the left cheek and necrotic skin infarct with irregular borders over the right cheek, chin, and forehead (black arrows).

**Figure 2:**
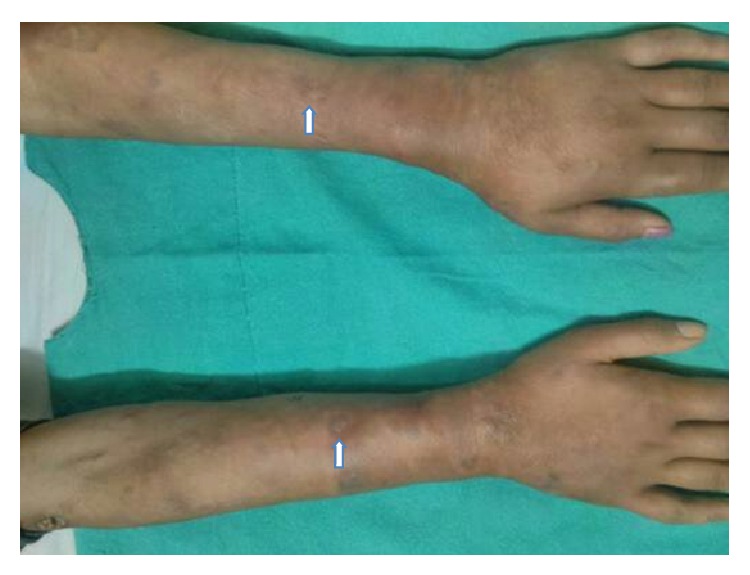
Picture of forearms and hands showing papulonodular infiltrating erythematous lesions over the forearms and dorsum of hands (white arrows).

**Figure 3:**
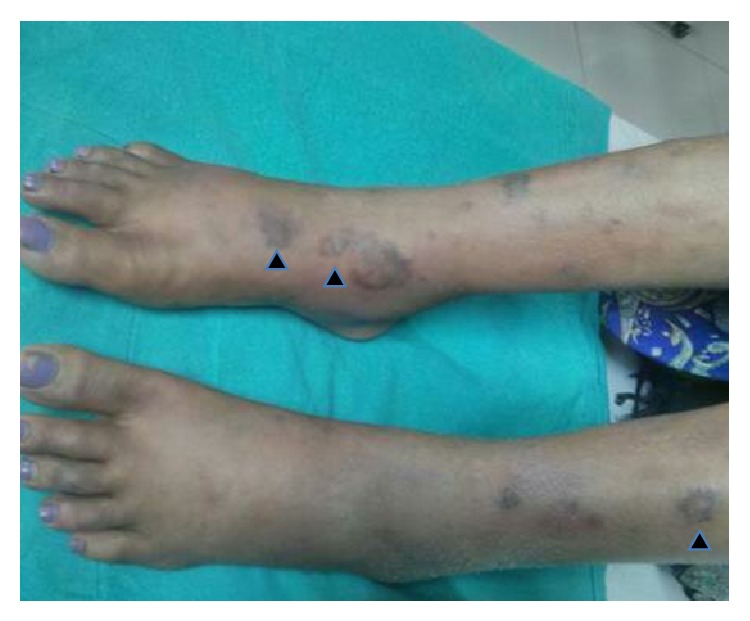
Picture of legs showing papules and nodules on dorsum of legs, necrotic lesions with irregular borders over lower leg and feet, and dorsal tenosynovitis of both feet (black arrowheads).

Examination revealed a temperature of 98°F, pulse rate of 98/minute with symmetry of all peripheral pulses, and blood pressure of 110/80 mm Hg in the right upper limb. There was mild pallor. She had multiple elevated plaque to nodule-like tender rashes, 1–3 cm in diameter, over arms, trunk, and upper and lower limbs (Figures 1, 2, and 3). The rashes over the face and both legs were necrotic, with black discolouration of the surface but no discharge or ulceration. She had bilateral axillary lymph nodes in the central group, 1 × 1 cm in size, discrete, nontender, and freely mobile. Musculoskeletal exam revealed extensor tenosynovitis over both feet (Figure 3); neurologic exam revealed thickening of both common peroneal and right ulnar nerves; however there was no tenderness or sensory impairment. There was an anaesthetic patch of 7 cm × 6 cm size with loss of sweating and appendages over the back. Systemic examination was otherwise unremarkable. Investigations revealed Hb 12.6 g%, microcytic and normochromic, TLC 16300/mm^3^, DLC showing neutrophils 80%, lymphocytes 15%, platelet count 463000/mm^3^, serum creatinine 0.8 mg%, serum alanine aminotransferase 28 U/L, serum bilirubin 0.7 mg%, serum lactate dehydrogenase mildly elevated (471 mg%, normal less than 450), and normal serum creatinine (0.8 mg%). Chest radiograph and urine examination were normal.

Such a clinical picture was consistent with Lucio phenomenon; however it was unusual for the same to occur so many months after starting antileprotic therapy. Also the cutaneous infarcts had occurred in spite of being on high dose steroids and antileprotic therapy for the past 4 months. There was no histologic evidence of leprosy until now, and medicines had been started based on a clinical diagnosis. So other differential diagnoses were considered, namely, cutaneous polyarteritis nodosa (fever, skin nodules, and skin infarcts with elevated ESR, neutrophilic leukocytosis, and thrombocytosis), cutaneous T-cell lymphoma (fever, subacute onset of skin rash, and poor response to steroids) and lupus profundus (fever with tender nodular skin rash affecting trunk and face; odd was skin infarcts).

**Figure 4:**
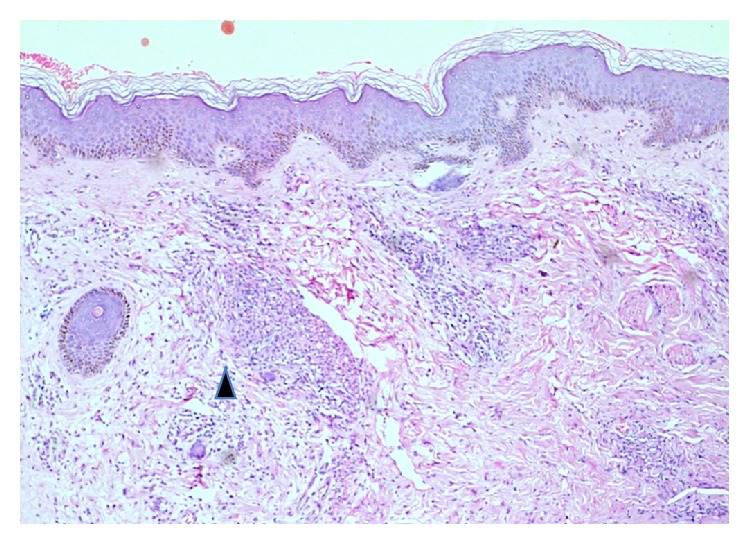
Skin biopsy from leg (hematoxylin and eosin stain, 20X magnification) showing largely unremarkable epidermis. Dermis shows collection of foamy histiocytes (black arrowhead).

**Figure 5:**
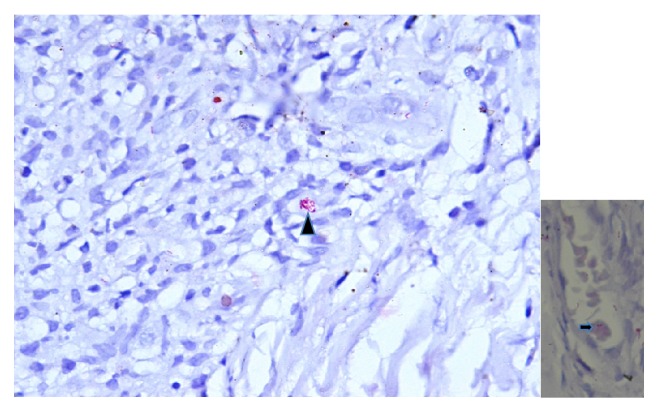
Magnified view of dermis showing foam cells with collection of lepra bacilli (globi) (black arrowhead) (Wade-Fite stain, 100X magnification); inset shows infiltration of capillary wall with lepra bacilli (black arrow) suggestive of Lucio phenomenon.

A skin biopsy was done to facilitate differential diagnosis. It showed unremarkable epidermis, foam cells with numerous lepra bacilli in dermis, and dermal capillaries showing vasculitis with neutrophilic infiltrate and damage to capillary wall with invading lepra bacilli (on Wade-Fite stain) (Figures 4 and 5), consistent with lepromatous leprosy with Lucio phenomenon. She was continued on prednisolone at 45 mg/day, with planned taper after 6 weeks, and rifampicin, clofazimine, and dapsone at the doses she was previously on (planned to be given for 24 months as per World Health Organisation recommendation for treating multibacillary leprosy). In addition, thalidomide was added at a dose of 100 mg daily to help with the lepra reaction. On OPD follow-up after 5 months, she was on prednisolone 10 mg/5 mg alternate day and continuing thalidomide 100 mg/day with antileprotic therapy as before. Her skin lesions and skin infarcts had healed and tenosynovitis and fever had resolved. ESR had normalized (13 mm/hour).

## 3. Discussion

Immunologic reactions in the context of leprosy can be of two types. Type I lepra reaction occurs on a background of tuberculoid leprosy, where cell-mediated immunity is robust, and is characterized by inflammation occurring inside existing skin lesions as well as appearance of new nodules and skin infiltrates. Type II lepra reaction, called ENL, occurs in lepromatous or borderline spectra, where cell-mediated immunity is weak and bacillary load is usually high. A rare form of lepra reaction is Lucio phenomenon, which manifests as tender nodules with ulceration, bulla formation, and necrotic areas [7–11]. Our patient had lepromatous leprosy with Lucio phenomenon.

What was odd in our patient for Lucio phenomenon was the onset of skin infarcts 4 months after starting antileprotic therapy. Lucio phenomenon is usually the presenting feature that heralds a diagnosis of leprosy [8, 12]. Also, the presence of cutaneous infarcts in the absence of blistering or ulcerating lesions is distinctly unusual for Lucio phenomenon (Magaña et al. reported a similar finding in only 3 out of 12 patients with Lucio phenomenon) [8]. Hence we considered differential diagnoses of cutaneous vasculitis or necrotising erythema nodosum. The skin biopsy was conclusively in favour of Lucio phenomenon occurring on a background of lepromatous leprosy and helped guide subsequent appropriate therapy, namely, continuing antileprotic therapy and prednisolone as well as addition of stronger immunosuppression with thalidomide. Our patient made a good recovery with this regimen.

Leprosy mimicking vasculitis has been rarely reported [9, 13–15]. Often the picture is complicated by the presence of autoantibodies as rheumatoid factor, antinuclear antibodies, and antineutrophil cytoplasmic antibodies. The pathology in Lucio phenomenon shows foam cells with lepra bacilli demonstrable inside them, as well as a cutaneous vasculitis involving medium and small-sized vessels [11]. Lucio phenomenon per se is common in Mexico and has only been rarely reported from India [16–20].

It is important for the clinician to differentiate leprosy from other presentations of cutaneous vasculitis, as the former is eminently curable with antibiotics and prudent use of immunosuppressive agents. A general principle is to always keep infectious etiologies in the differential diagnosis of vasculitis, as the treatment for the two is dramatically different and inappropriate immunosuppression alone can be disastrous in the context of infection. Leprosy is gaining attention as a global health problem due to reactivation of latent, previously undiagnosed cases even in the western world due to use of strong immunosuppressive regimens for a variety of diseases [1, 2]. When in doubt, a skin biopsy often helps to get the final diagnosis.
